# Chemical properties of superatomic Li_3_O clusters from a density functional theory perspective: formation of chloride and adsorption behavior on graphynes†

**DOI:** 10.1039/d3cp05478k

**Published:** 2024-03-19

**Authors:** Xiao Wang, Meng Zhang, Wei Cao

**Affiliations:** a School of Physics, East China University of Science and Technology Shanghai 200237 China mzhang@ecust.edu.cn; b Nano and Molecular Systems Research Unit, University of Oulu FIN-90014 Finland wei.cao@oulu.fi

## Abstract

Superatomic clusters have received a lot of attention due to their ability to mimic the electronic configurations of individual atoms. Despite numerous studies of these clusters, their ability to mimic the chemical properties of individual atoms is still unclear. This also applies for Li_3_O/Li_3_O^+^ clusters which simulate the Na atom and its ion, but their capabilities to form a salt or be adsorbed on surfaces remain unexplored. In this work, a density functional theory investigation was performed to study the chemical formation and adsorption behavior of the superatomic Li_3_O cluster. The results show that Li_3_O mimics the chemical properties of the sodium element to form Li_3_O chloride and be adsorbed on graphdiyne and γ-graphyne with similar binding energy as the sodium adsorbate cases. Beyond the isolated cluster individuals, superatoms are demonstrated as elements from the 3D periodic table to construct compounds and attach onto solid surfaces.

## Abstract 2

The superatom identity of the Li_3_O cluster is studied by density functional theory. Li_3_O can mimic a Na atom in the electronic configuration and maintain this mimicry when bonding with halogen element atoms and adsorbing on graphynes.

## Graphical abstract

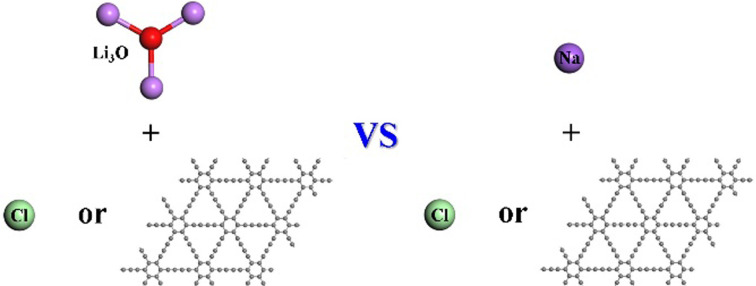

## 1. Introduction

One of the breakthroughs in cluster research was the discovery of superatoms. With appropriate size and composition, these specific clusters have similar electronic structures to those of atoms in the periodic table. If superatoms are placed into the same positions as their master atoms, a three-dimensional (3D) periodic table can be constructed^1^ where superatoms are piled up above their masters. Detected in gaseous or free form, the superatoms are labeled by more conspicuous peaks compared to those from neighbouring isomers. The experimental observations imply more abundance and better stability of the superatomic clusters compared with their peers.^2,3^ As a result, they are considered as building blocks for new synthetic materials. Additionally, in molecules composed of superatomic clusters, the (sub)cluster structures may be kept and enrich potential functionalities originating from component clusters. Hence, molecules formed by superatoms are considered to own superior functionalities over molecules directly formed by atoms.^4–8^

Over the past twenty years, many theoretical and experimental studies have been conducted to create superatoms and explore their structures and properties. So far, a variety of superatoms, including superalkalis,^9–11^ superalkaline-earth-metal atoms,^12^ superhalogens,^13–15^ multivalent superatoms,^16,17^ and magnetic superatoms^18–20^ have been designed and identified using different electron-counting rules such as the jellium rule, octet rule and Wade–Mingos rule *etc.* These research studies further interpretate the concept of a superatom and provide a variety of structural building blocks for cluster-assembled materials (CAMs). For example, C_60_ can form an FCC solid through van der Waals force at room temperature and pressure.^21–23^ An alkali-like superatom TaSi_16_ can combine with C_60_, resulting in a superatomic complex with high thermal and chemical robustness.^24,25^ Other CAMs, such as ZnO-based CAMs, Al-based CAMs and zintl cluster-based CAMs, have also been synthesized and investigated.^26–31^ In fact, clusters can be assembled on substrates as well as in confined spaces. A proper substrate is the key to host clusters and preserves their superatomic identities. For instance, weak interactions between the FeCa_8_ superatom and the h-BN or graphene can maintain the structure and characteristics of the superatomic cluster. On the contrary, the strong interaction between the FeCa_8_ and a calcium substrate leads to a destruction of the magnetic properties of the cluster.^32^ Therefore, it is important to understand the interactions between superatomic clusters and their ligands or hosting matrices, and the interactions’ impacts on the structures and properties of bonded or adsorbed systems.

Among the studied superatoms, the structure and properties of superalkali atom Li_3_O in its neutral or charged form have been systematically investigated.^33–36^ In our previous work,^37^ the predicted Li_3_O^+^ has been identified in the gas form. Its formation process has been proposed and the possibility of application as a building unit for energy storage has been uncovered. It is further noticed that Li-rich antiperovskite (LiRAP) conductors Li_3_OA (A = halogen or superhalogens) with lithium superionic conductivity have been created and considered to have great promise for solid-state electrolytes.^38–40^ Recent first-principles studies of Li_3_O functionalized graphyne, graphdiyne and h-BN sheets indicated that superalkali clusters can inhibit metal agglomeration. Thanks to facilitated charge transfers, the cluster decorated sheets are found to be suitable for hydrogen storage.^41,42^ Despite the above advances, the superatomic cluster's bonding schemes with elements and interactions with matrices are far less understood compared to knowledge of the structural and electronic configurations of individual clusters. The chemistry of superatomic clusters (including the Li_3_O) remains elusive.

In this work, we carried out a density functional theory study to investigate the physical and chemical properties exhibited by Li_3_O clusters when interacting with ligands or substrates. At the free form level, the Li_3_O cluster can mimic a single Na atom in electronic configuration. It also mimics the bonding or interaction schemes of the Na when bonding with a halogen atom or being adsorbed on two-dimensional graphynes. A chemical bond is formed between the superatomic Li_3_O cluster and chlorine, and the adsorption energies of Li_3_O onto the graphynes are almost the same as the case using Na adsorbate. From the above results, it is hoped that this work will provide a theoretical reference for further application of these clusters in compound formations and surface interactions.

## 2. Computational methods

All computations in this work were performed through density functional theory (DFT) calculations implemented in the Cambridge Serial Total Energy Package (CASTEP) package.^43^ They are detailed in the geometric optimization and electronic structure of the pristine Li_3_O superatom, the cluster's interaction with halogen elements, and adsorption on the graphdiyne (GDY) and γ-graphyne (γ-GY). The exchange–correlation was treated with the generalized gradient approximation (GGA) in the form of the Perdew–Burke–Ernzerhof (PBE) functional together with the employment of OTFG ultra soft pseudopotentials.^44^ Koelling–Harmon was applied as a relativistic effect treatment and spin polarization was chosen during the geometric optimization. A value of 10^−6^ eV per atom was set for the total energy SCF convergence threshold, and 450 eV as the cut-off energy to expand the plane waves included in the basis set. The quality of the Monkhorst–Pack *k*-points was specified on 3 × 3 × 1 for geometry optimization and 6 × 6 × 1 *k*-point grid for the calculation of density of states. Our calculations were carried out using 3 × 3 graphynes supercells with periodic boundary conditions in the basal plane to simulate infinite planar sheets. A vacuum region of 20 Å was applied in the *z*-direction to ensure negligible interaction between adjacent layers.

The binding/adsorption energies of the Na atom or Li_3_O cluster interacted with other Cl/F or GDY/γ-GY systems were computed as follows.

1 *E* = *E*_total_ − [*E*(*X*) + *E*(*Y*)]

where *E*_total_ is the energy for the complex, and *E*(*X*) and *E*(*Y*) represent the total energy of the lowest-energy structures of pristine Na/Li_3_O and Cl/F/GDY/γ-GY, respectively.

## 3. Results and discussion

### 3.1 Characteristics of the Li_3_O cluster

**Fig. 1:**
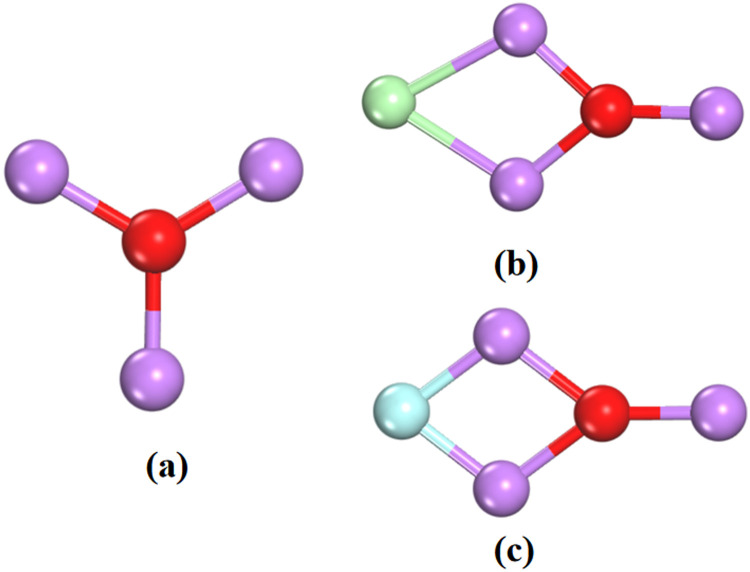
The lowest energy structures of (a) the superatomic Li_3_O cluster, (b) (Li_3_O)Cl, and (c) (Li_3_O)F halides. Lithium, oxygen, chlorine and fluorine atoms are represented in violet, red, green and blue, respectively.

Following geometry optimization, the most stable Li_3_O cluster owns a highly symmetric *D*_3h_ triangle structure with the oxygen atom in the center as displayed in Fig. 1(a). The bond length between the lithium and oxygen atoms is 1.702 Å, in line with the reported value from Yokoyama *et al.*^45^ An Li_3_O cluster has nine valence electrons coming from Li and O sites: each Li atom contributes one valence electron, and the O atom contributes six valence electrons.

**Fig. 2:**
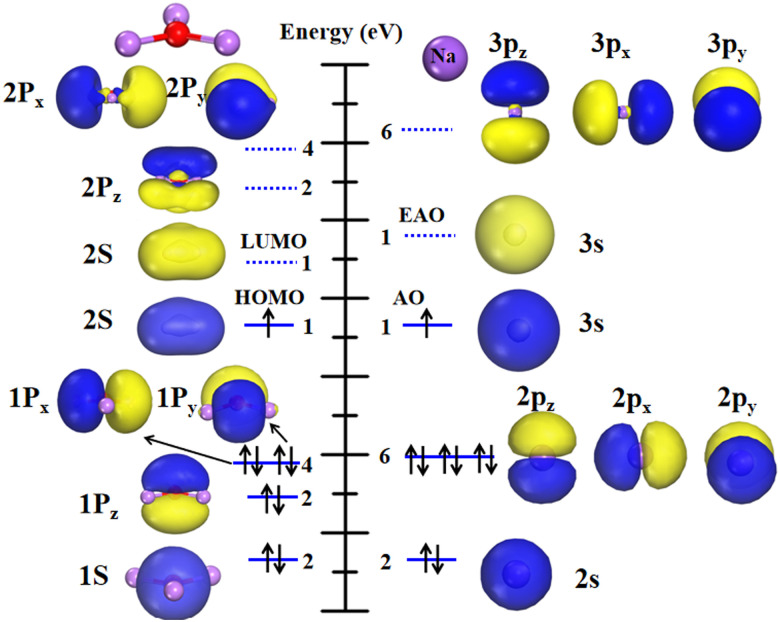
Relative energy levels and orbitals of the Li_3_O cluster (left) and Na atom (right). EAO represents the empty atomic orbital.

The molecular orbital (MO) characteristics of the superatom Li_3_O are illustrated in Fig. 2 and the atomic orbitals (AOs) of the sodium atom are also depicted for comparison. It is shown that the electronic structure of Li_3_O can be described as 1S^2^1P^6^2S^1^, which bears resemblance to that of an individual sodium atom. Obviously, it is apt to lose one electron to achieve a closed shell electron configuration of 1S^2^1P^6^, thus mimicking the chemical behavior of the alkali atoms. We further studied the electronic structure of the cluster in detail. The lowest valence MO of Li_3_O exhibits 1S character, followed by two degenerate 1P_*z*_ orbitals. The degenerate 1P_*x*_ and 1P_*y*_ orbitals are pushed 0.37 eV higher in energy owing to the planar structure of the Li_3_O cluster. This is slightly different from the sodium atom case where three degenerate p orbitals exist. These occupied MOs of Li_3_O are mainly composed of the AOs of the central atom O. Then, the following highest occupied molecular orbital (HOMO) of the cluster has a half-filled 2S superorbital character. Three 2P orbitals are the next higher energy orbitals. Similar to the case in 1P orbitals, 2P orbitals degenerate into two groups of one (P_*z*_) and two (P_*x*_ and P_*y*_) orbitals, as induced by the two-dimensional planar structure of the cluster. It should be noted that in Fig. 2, the electron cloud pervades the entire cluster. A shape difference stays between the cluster's 2S MO and the Na's 3s AO. The former appears flattened, while the latter is spherical. This discrepancy arises from the planar geometry of the Li_3_O. Therein, the delocalized electron distribution from each atom is confined around the 2D plane. An energy gap (*E*_gap_) of 0.631 eV was found between the highest occupied molecular orbital and the lowest unoccupied molecular orbital (HOMO–LUMO). Although the *E*_gap_ value of the Li_3_O cluster is smaller than that of the Na atom (1.633 eV), its chemical stability is approximate to that of the Na atom, which will be discussed in the following section.

### 3.2 Chemistry of Li_3_O

In order to examine the similarity between the Li_3_O cluster and the Na atom, the chemistry of the Li_3_O cluster is discussed by considering the interaction of the Li_3_O cluster with halogen atoms and adsorption on graphynes.

#### 3.2.1 (Li_3_O)Cl similar to NaCl

An important property of the Na atom is its violent reaction with halogen atoms to form salts through ionic bonds. For example, the Na atom can react with F, Cl, and I atoms to form the NaF, NaCl, and NaI salts. To examine the similarity between the Li_3_O and Na atom in forming salts, the above Li_3_O was bonded to the Cl atom to form the Li_3_O–Cl compound. The lowest energy geometry is displayed in Fig. 1(b).

After careful optimization, the triangle Li_3_O cluster interacts with a Cl atom through two Li atoms through an edge connection. The binding energy (BE) of Li_3_O to Cl is −5.718 eV. The bond length (distance between Li and Cl atom) is 2.268 Å, very close to the value of 2.373 Å in the NaCl. Table 1 shows that the natural charge on the Cl is - 0.613 |*e*|, indicating that almost one electron transfers from the Li_3_O cluster to the Cl atom to form an ionic bond. Each Li atom of Li_3_O contributes an average charge of 0.494 |*e*|, sharing with O and Li atoms. The electrostatic interaction between Li_3_O^+^ and Cl^−^ causes a slight variation in the average bond length of Li–O, which is elongated by 0.04 Å compared to the bare Li_3_O. All these observations indicate that the compound (Li_3_O)Cl is actually a ‘‘binary salt’’ consisting of Li_3_O^+^ and Cl^−^. The same ionic interaction is also observed in (Li_3_O)F which has a similar structure of (Li_3_O)Cl as shown in Fig. 1(c). The BE is −6.275 eV and the distance of the Li and F atom is 1.78 Å. The Li_3_O subunit carries 0.682 |*e*| net charge in (Li_3_O)F. Each Li atom transfers 0.516 |*e*| to O and F atoms. These characteristic data are also very close to those of NaF molecules. Given the above, it can be seen that Li_3_O is capable of combining with Cl or F atoms to form stable ionic compounds analogous to the sodium salts.

**Table 1:** The calculated binding energies of (Li_3_O)Cl, NaCl, (Li_3_O)F and NaF, the corresponding distance of the Cl (F) atom to Li_3_O (the nearest Li to Cl or F atom), the corresponding distance between Na and Cl (F) and the charge of Li_3_O (Na) and Cl (F)

System	Binding energies (eV)	Bond length	Charge (|*e*|)	
			Li_3_O/Na	Cl/F
(Li_3_O)Cl	−5.718	2.268	0.613	−0.613
NaCl	−4.319	2.373	0.713	−0.713
(Li_3_O)F	−6.275	1.78	0.682	−0.682
NaF	−5.251	1.94	0.74	−0.74

We also constructed the 3Li–O–Cl clusters with all atoms randomly distributed and without a predetermined geometric structure for Li_3_O. Extensive two dimensional (2D) and three-dimensional (3D) structures were constructed to determine the lowest-energy geometries for the 3Li–O–Cl clusters. The details of the metastable structures are depicted in Fig. S1 in the ESI.† During the geometric optimization process, the O atom and the three Li atoms spontaneously tend to assemble into a superatomic cluster structure before interacting with the Cl atom. In other words, the introduction of the highly reactive chlorine with strong chemical properties does not impact the formation of the Li_3_O superatomic cluster in its free state. Hence, the superatom Li_3_O is highly stable and can be assembled easily, consistent with our previous experimental findings based on time-of-flight mass spectrometry.^37^

**Fig. 3:**
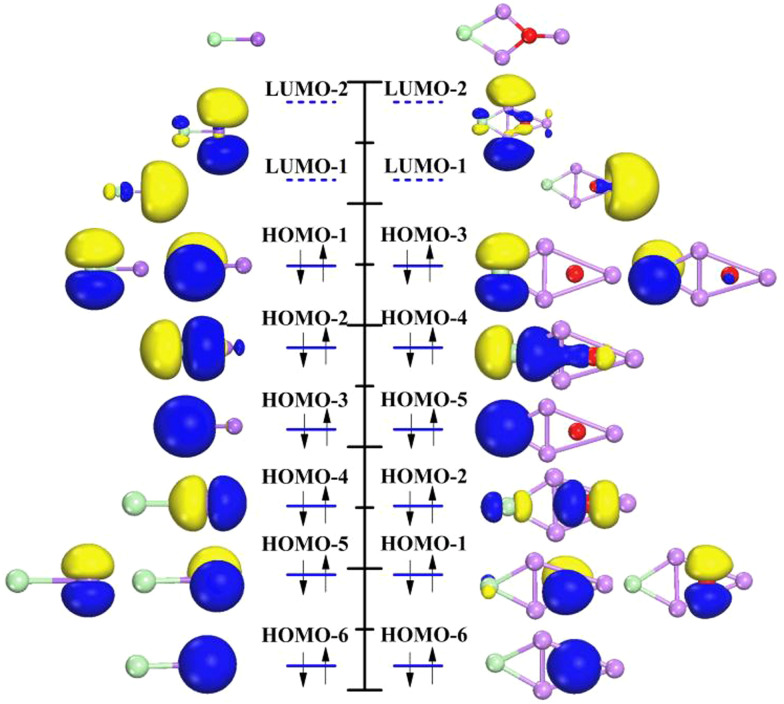
Molecular orbitals of the NaCl (left) and (Li_3_O)Cl (right).

Next, the molecular orbitals of (Li_3_O)Cl and the corresponding NaCl are investigated and shown in Fig. 3. Since the main intention here is to provide a comparison of the orbital shapes between two kinds of compounds, the orbitals in Fig. 3 are arranged in the order of energy without strictly adhering to their exact energy positions. In NaCl, the Na 3s orbital combines with Cl 3p_*z*_ to form molecular orbitals labeled as 4σ and 4σ* in Fig. 3 (left). They are named as HOMO−2 and LUMO−1. The 4σ orbital is weakly bonded and mostly Cl-like in character. The antibond 4σ* orbital is centered almost entirely on the Na atom. HOMO−1 comes from Cl 3p_*x*_ and 3p_*y*_ which could be labeled as 2π orbitals and displayed as HOMO−1. The electronic configuration of (Li_3_O)Cl is analogous to that of NaCl. It is obvious that the superatomic S and P orbitals behavior is similar to the s and p AOs of the Na atom. The σ, σ* and π orbitals of (Li_3_O)Cl are also analogous to the σ, σ* and π orbitals of NaCl, respectively. Two molecular HOMOs are similar to σ* orbitals indicating that the two molecules have some chemical similarities. This finding is in line with previous studies of the halogen-like superatom Al_13_ and chlorine atom, superatom B_6_ and Ni atom. Although there are some variations in orbital order that occur in the case of bonding with the Cl atom, the similarity between the Li_3_O and Na is still exhibited.

#### 3.2.2 Li_3_O adsorption on graphynes

**Fig. 4:**
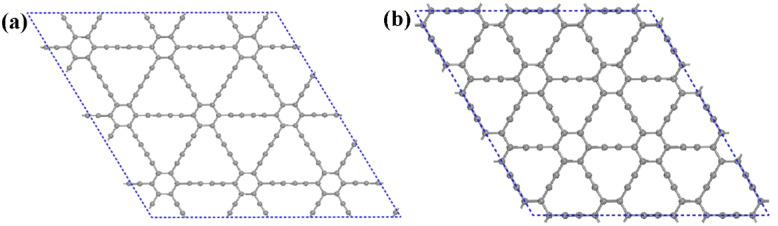
The optimized structures of (a) GDY and (b) γ-GY.

After examining the chemical bonding between Li_3_O and the Cl atom, we also studied the interaction between Li_3_O and graphynes. Here, we take Li_3_O adsorption on GDY and γ-GY as examples, following the Li-cluster's roles in 2D energy storage materials.^46^ First, supercells of the pristine GDY and γ-GY nanosheets are fully relaxed without any constraints. As shown in Fig. 4, the optimized primitive structures of a GDY layer and γ-GY layer have a lattice constant of *a* = *b* = 9.450 Å and *a* = *b* = 6.953 Å, respectively. The values are in a good agreement with previous reports.^47–49^ Unlike graphene, which only contains sp^2^ hybridized carbon atoms, GDY and γ-GY are composed of sp and sp^2^ hybridized C atoms. In GDY, there are two triple bonds in the carbon link (–C≡C–C≡C–), while the linkage between two neighboring benzene rings is the –C≡C– carbon link in γ-GY.

**Fig. 5:**
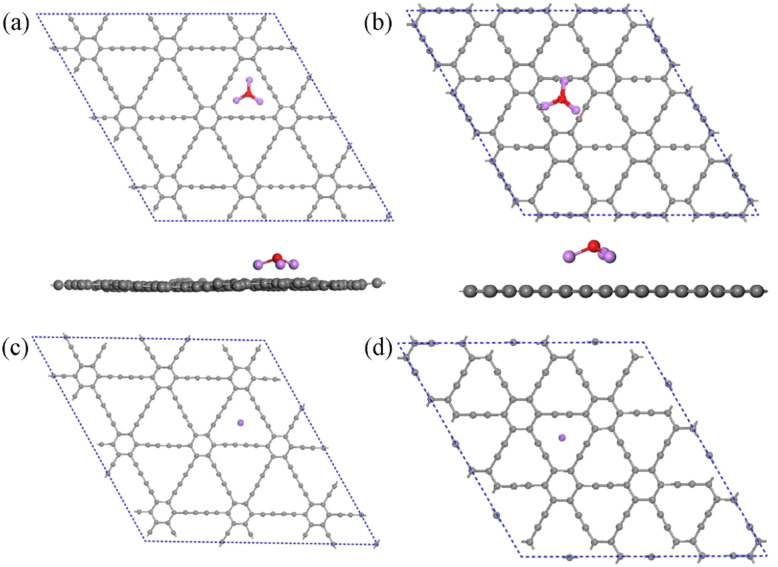
The optimized structures of Li_3_O adsorption on (a) GDY and (b) γ-GY. The optimized structures of Na atom adsorption on (c) GDY and (d) γ-GY.

The geometrical structures of Li_3_O cluster adsorption on GDY and γ-GY are systematically studied. First, all possible adsorption sites are considered, including the hollow sites above the center of the carbon rings, the top site right above a carbon atom and the bridge site above the carbon bonds. After careful structural optimization, the most stable configurations of the Li_3_O decorated GDY and γ-GY are obtained and displayed in Fig. 5. In the complex of Li_3_O@GDY, the Li_3_O unit prefers to stay above the center of the triangle pore formed by the –C≡C–C≡C– linkages. An adsorption energy of −3.70 eV is obtained and listed in Table 2, which is a little lower than the previous study.^42^ The shape of the Li_3_O cluster changes from a triangle to an umbrella where the plane formed by three Li atoms is parallel to the plane of GDY. The Li–O bond length increases to 1.71 Å after absorption. Meanwhile, the bond angles of Li–O–Li reduced to 113° from 120° in the pristine cluster. Li_3_O also prefers to bind on the hollow site of the C_12_-ring due to it having the largest binding energy of −3.56 eV in the Li_3_O@γ-GY case. The bond length of Li–O is 1.72 Å and the Li–O–Li angle changes to 107°. Additionally, the binding energy between Li_3_O and GDY is larger than that between Li_3_O and γ-GY. This is because each Li atom of the Li_3_O unit binds with four sp hybridized carbon atoms in the Li_3_O@GDY configuration. Due to the chemical activity of the triple carbon bonds of the GDY, the top site of the acetylenic ring is often the most stable adsorption site for single metal atom adsorption. However, according to the previous works,^50–53^ the atoms would automatically migrate to the corner when the transition metal (TM) or noble metal atoms were located at the center of the acetylenic ring. While alkali metals such as Li, Na and K would be anchored at the triangle center of GDY.^54^ These are in line with the adsorption of Na on the GDY and γ-GY, as depicted in Fig. 5(c) and (d). In addition, from Table 2, the adsorption energy of the Na atom on GDY or γ-GY is almost the same as the corresponding one of Li_3_O on GDY or γ-GY.

**Table 2:** The calculated adsorption energies of Li_3_O@graphynes and Na@graphynes, and the average charge or net charge of the complexes and their individual atoms

System	Adsorption energies (eV)	Charge (|*e*|)			
		Li atoms	O atom	Li_3_O/Na	Carbon sheet
Li_3_O@GDY	−3.70	1.35	−0.84	0.51	−0.51
Na@GDY	−3.71			0.84	−0.84
Li_3_O@GY	−3.56	1.50	−0.87	0.63	−0.63
Na@GY	−3.18			0.80	−0.80

**Fig. 6:**
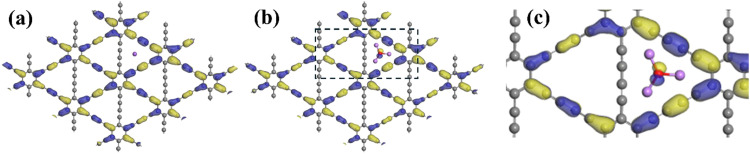
SOMO orbital diagram of the (a) Na@GDY, (b) Li_3_O@GDY and (c) zoomed-in region next to Li_3_O on (b).

To better understand and compare the binding mechanism of these complexes, we studied the electronic structures of Na@GDY and Li_3_O@GDY as typical cases. The Na atom donates a single valence electron in the 3s orbital to the LUMO of GDY. Consequentially, a singly occupied molecular orbital (SOMO) is formed in Na@GDY. It is composed of p orbitals perpendicular to the GDY plane as shown in Fig. 6. Li_3_O@GDY has a similar intramolecular charge transfer mechanism. Due to the low ionization potential, the Li_3_O cluster donates its electrons to the acetylenic linkages of the GDY sheets and these electrons occupy the empty p_*z*_ orbitals of the carbon frame. It is worth noting that Fig. 6 shows inconsistent electron clouds around the adsorbed Na on Na@GDY and Li_3_O on Li_3_O@GDY. While the Na atom does not exhibit obvious electron density in the center of the triangle, the adsorbed superatom behaves differently. The electron clouds are centered next to the oxygen and keep a similar P-type distribution as in the free cluster (Fig. 2) or in ionic bonds (Fig. 3). However, almost no electron cloud could be detected on the Li, denoting the charge transfer from the Li to neighboring atoms. A zoomed-in image of the adsorbed structure in Fig. 6(c) further denotes slight increase of the electron densities on the C atoms next to the Li atoms. This proves that the charge transfer happens from Li_3_O to the GDY after absorption. In other words, the inner shell electronic structure is kept within the cluster to keep the superatomic feature, but the valence electron from 2S is involved in charge transfer in the adsorption.

Furthermore, the bandgap energy of the adsorbed system is investigated. The pristine GDY is a semiconductor with a bandgap of 0.483 eV. However, both Na@GDY and Li_3_O@GDY exhibit metal behavior after decorations of the Na or Li_3_O. The bandgap energy decreases due to the redistribution of electrons following intramolecular charge transfers. Similar phenomena have also been reported in TM adsorbed GDY/GY due to electron transfers between the TM adatoms and the GDY/GY.^50,51^

**Fig. 7:**
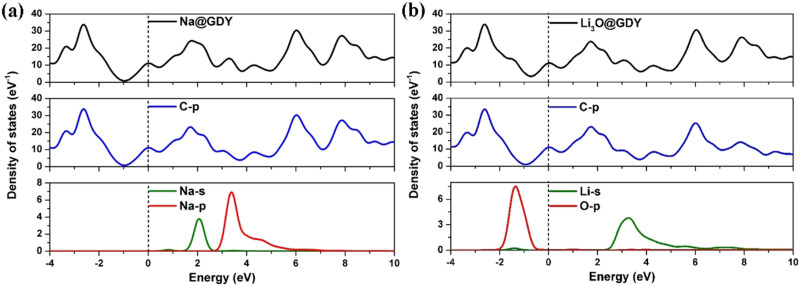
The PDOS of the (a) Na@GDY and (b) Li_3_O@GDY. The Fermi level is denoted by a vertical dashed line.

The partial density of states (PDOS) of the Na@GDY and Li_3_O@GDY are further investigated and illustrated in Fig. 7. It can be observed that the PDOS shapes of both systems are quite similar. The s and p orbitals of Na atoms, as well as s orbitals of Li in the Li_3_O clusters, exhibit pronounced hybridization with C-2p orbitals of the GDY system around 2–6 eV. This suggests that both Na atoms and Li_3_O clusters can interact with GDY stably. The Li_3_O cluster adsorbed on GDY is comparable to that of a single Na atom adsorbed on GDY. However, in Li_3_O@GDY, besides the significant hybridization between the C-2p orbitals of GDY and the s orbitals of Li in the 2.5–6 eV range, the PDOS of Li below the Fermi level overlaps with O-2p. The overlaps indicate strong bonding between Li and O and denote high stability of the Li_3_O superatom after absorption. This is different from the case in Na@GDY where no overlap is observed at a similar energy range.

## 4. Conclusion

To summarize, the superatom identity of the Li_3_O cluster has been systemically studied using DFT in this work. A Li_3_O cluster can easily lose one electron to achieve a closed shell electron configuration of 1S^2^1P^6^. It bears a great resemblance to the Na atom in the electronic configurations and chemical properties to form chloride and adsorption on graphynes. Li_3_O is able to form ionic bonds with chlorine or fluorine element and the molecular orbitals of (Li_3_O)Cl are analogous to those in the sodium salts. We also compared adsorptions of the Li_3_O cluster and Na atom on graphdiyne and γ-graphyne. Similar to the Na atom, the Li_3_O cluster preferentially interacts with the large triangular hole in extensively delocalized π-conjugated GDY and γ-GY. Interestingly, the adsorption energies are found to be the same for the Li_3_O@GDY and Na@GDY, and very close in the Li_3_O@GY and Na@GY cases. Meanwhile, the bandgaps of GDY and GY after adsorbing either superatom Li_3_O or Na atom reduce to zero due to the intramolecular charge transfer. The effects of doping Li_3_O into the surface of GDY on the electronic structures were further investigated. Electrons transfer from the Li_3_O cluster to the GDY sheets and occupy its empty p_*z*_ orbitals, similar to an electron from Na to the GDY. In conclusion, Li_3_O can combine with halogen elements and interact with graphynes as a superatom counterpart of the sodium element.

## Conflicts of interest

There are no conflicts to declare.

## Supplementary Material

CP-026-D3CP05478K-s001

CP-026-D3CP05478K-s002
