# Supplementary material for: Chemical properties of superatomic Li3O clusters from a density functional theory perspective: formation of chloride and adsorption behavior on graphynes
Source: Phys Chem Chem Phys. 2024 Mar 19;26(15):11708–14. doi: 10.1039/d3cp05478k (PMC11022547; doi:10.1039/d3cp05478k)
Supplement: CP-026-D3CP05478K-s002 [file CP-026-D3CP05478K-s002.pdf]

Electronic Supplementary Material (ESI) for Physical Chemistry Chemical Physics.
This journal is © the Owner Societies 2024

Supplementary Information for

Chemical properties of superatomic Li_3O clusters from density functional theory perspective: formation of chloride and adsorption behavior on graphynes

Xiao Wang[1], Meng Zhang[1], Wei Cao[2]

[1] School of Physics, East China University of Science and Technology, Shanghai 200237, China.

[2] Nano and Molecular Systems Research Unit, University of Oulu, FIN-90014, Finland.

Correspondence:

Meng Zhang, Email: mzhang@ecust.edu.cn

Wei Cao, Email: wei.cao@oulu.fi

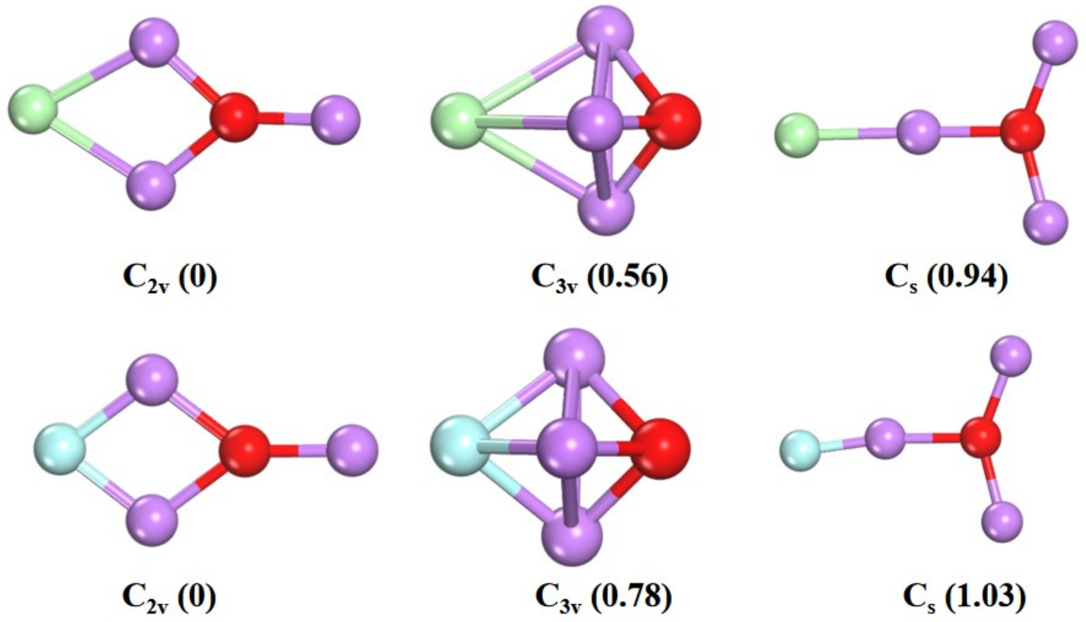

Figure S1. The lowest energy structures and the metastable isomers of the $Li_3O(Cl)$ and $Li_3O(F)$ halides. Numbers in the brackets are the energies (in eV) of the clusters relative to the lowest energy energies. Green, blue and red spheres represent chlorine, fluorine and oxygen atoms, respectively. Space groups were determined with a tolerance of 0.1 Å.
